# In Parkinson’s disease dopaminergic medication and deep brain stimulation of the subthalamic nucleus increase motor, but not reflection and cognitive impulsivity

**DOI:** 10.3389/fnins.2024.1378614

**Published:** 2024-06-19

**Authors:** Martijn Hendriks, Saman Vinke, Rok Berlot, Mitja Benedičič, Marjan Jahansahi, Maja Trošt, Dejan Georgiev

**Affiliations:** ^1^Department of Neurology, University Medical Centre Ljubljana, Ljubljana, Slovenia; ^2^Department of Neurosurgery, Donders Institute for Brain, Cognition and Behaviour, Radboud University Medical Centre, Nijmegen, Netherlands; ^3^Faculty of Medicine, University of Ljubljana, Ljubljana, Slovenia,; ^4^Department of Neurosurgery, University Medical Centre Ljubljana, Ljubljana, Slovenia; ^5^Department Clinical and Motor Neurosciences, Institute of Neurology, University College London, London, United Kingdom; ^6^Artificial Intelligence Laboratory, Faculty of Computer and Information Science, University of Ljubljana, Ljubljana, Slovenia

**Keywords:** deep brain stimulation, dopaminergic medication, motor impulsivity, cognitive impulsivity, reflection impulsivity, Parkinson’s disease

## Abstract

**Background:**

Parkinson’s disease is associated with increased impulsivity, which can be divided into several domains: motor (consisting of proactive and reactive subdomains), reflection, and cognitive impulsivity. Evidence suggests that both dopaminergic medication and subthalamic nucleus deep brain stimulation can affect impulsivity. Therefore, we set out to investigate the effects of dopaminergic medication and subthalamic nucleus deep brain stimulation on motor, reflection, and cognitive impulsivity in Parkinson’s disease patients.

**Methods:**

Twenty Parkinson’s disease patients who underwent subthalamic nucleus deep brain stimulation were tested ON and OFF dopaminergic medication and ON and OFF subthalamic nucleus deep brain stimulation. They performed three different impulsivity tasks: the AX continuous performance task (AX-CPT) to test for motor impulsivity, the Beads task for reflection impulsivity, and the Delay discounting task for cognitive impulsivity.

**Results:**

The combination of subthalamic nucleus deep brain stimulation and dopaminergic medication led to an increase in motor impulsivity (*p* = 0.036), both proactive (*p* = 0.045) and reactive (*p* = 0.006). There was no effect of either dopaminergic medication or subthalamic nucleus deep brain stimulation on reflection and cognitive impulsivity.

**Conclusion:**

The combination of dopaminergic medication and subthalamic nucleus deep brain stimulation leads to increased motor, but not cognitive or reflection, impulsivity in patients with Parkinson’s disease. Both proactive and reactive motor impulsivity were impaired by the combination of dopaminergic medication and subthalamic nucleus deep brain stimulation.

## Introduction

Patients with Parkinson’s disease (PD) often develop cognitive impairments and executive dysfunction in addition to the most common motor symptoms such as bradykinesia, tremor, rigidity, and impairments of postural reflexes (Dubois and Pillon, 1996; Dirnberger and Jahanshahi, 2013). While previous research suggests that dopaminergic medication (Armstrong and Okun, 2020) and subthalamic nucleus deep brain stimulation (STN-DBS) effectively control the motor symptoms of the disease (Deuschl et al., 2006; Fasano et al., 2012; Weaver et al., 2012), there is evidence for impaired executive functions with dopaminergic medication (Cools, 2001) and STN-DBS in patients with PD (Fasano et al., 2010). One of the most widely explored behavioral changes related to dopaminergic medication or STN-DBS is impulsive behavior, which even though a unitary phenomenon, can be distinguished into multiple domains depending on the context of testing. *Cognitive impulsivity*, also referred to as an impulsive choice (Dalley Jeffrey et al., 2011), is described as a tendency to make rash decisions without effective evaluation of alternative choices (Cáceres and San, 2017) and can be expressed as impaired decision-making (Antonelli et al., 2011). *Reflection impulsivity* refers to reacting quickly without pausing for reflection (Jahanshahi, 2013). *Motor impulsivity*, on the other hand, refers to the impaired inhibition of a previously learned response and can be reactive or proactive (Antonelli et al., 2011). Reactive motor impulsivity refers to the inability to stop a response when a specific stop-signal is indicated (Antonelli et al., 2011). Proactive motor impulsivity is more goal-directed and relates to self-control. It involves, methods cautious responding to meet goals and objectives (Jahanshahi, 2013).

Dopaminergic medications have been associated with changes in all areas of impulsivity in PD: motor (Servan-Schreiber, 1996; Cohen et al., 1999; Nombela et al., 2014; Canário et al., 2019), reflection (Huq et al., 1988; Djamshidian et al., 2013), and cognitive (Antonelli et al., 2014; Canário et al., 2019) impulsivity. With STN-DBS in PD, there is evidence that DBS can increase impulsive responding in conditions of high decision conflict (Georgiev et al., 2016), while dopaminergic medication had no effect on this form of impulsivity (Frank et al., 2007). Another study examining the processing of rewards found that STN-DBS increased risky choices but did not worsen the evaluation of delayed rewards (Evens et al., 2015). Furthermore, it appears that, compared to healthy controls, STN-DBS in PD may increase reactive but not proactive impulsivity (Obeso et al., 2013). There is also evidence that dopamine agonists, rather than STN-DBS, increase reflection impulsivity (Djamshidian et al., 2013). Therefore, the results of the studies examining the effect of dopaminergic medication and STN-DBS on the different facets of impulsivity are inconsistent. To the best of our knowledge, to date, no study has tested the effect of both dopaminergic medication and STN-DBS within the same patient group on all three major impulsivity domains. Therefore, we set out to test the effect of STN-DBS and dopaminergic medication on motor, reflection, and cognitive impulsivity in PD patients treated with STN-DBS.

## Methods

### Subjects

Twenty consecutive PD patients were enrolled in the study. All PD patients met the Queen Square Brain Bank criteria (Gibb and Lees, 1988) for the diagnosis of PD. All patients underwent surgery using the MRI-guided approach in combination with intraoperative microelectrode recording and intraoperative testing and had been under stable STN-DBS treatment for at least 4 months at the time of recruitment. Patients were recruited from the outpatient clinics by two movement disorder specialists (DG and MT) from the Department of Neurology at the University Medical Centre in Ljubljana. The study was approved by the Medical Ethical Committee of the Republic of Slovenia (number 0120–503/2016/6).

### Clinical assessment

Cognition was assessed using the Montreal Cognitive Assessment (MoCA) (Nasreddine et al., 2005). The Beck Depression Inventory-II (BDI-II) (Beck et al., 1996) was used to assess the presence and severity of depression. Scores from 0 through 13 indicated no or minimal depression; scores from 14 through 19 indicated mild depression; scores from 20 through 28 indicated moderate depression; and scores from 29 through 63 indicated severe depression (Beck et al., 1996). The presence of apathy was assessed using the Starkstein Apathy Scale (SAS) (Starkstein et al., 1992). Patients with SAS values of 14 and more were considered apathetic (Starkstein et al., 1992). The total score and the second-order subscales (attentional, motor, and non-planning) of the Barratt Impulsiveness Scale 11 (BIS-11) (Patton et al., 1995) were used to assess trait impulsivity in patients. Patients with a total BIS-11 score of 72 and above were considered highly impulsive. Patients with scores between 52 and 71 were considered within the normal range of impulsiveness (Stanford et al., 2009). The Questionnaire for Impulsive-Compulsive Disorders in Parkinson’s Disease-Rating Scale (QUIP-RS) (Weintraub et al., 2012) was used to assess the presence of impulse control disorders in patients. The established cut-offs used to determine the presence of certain impulsive behaviors based on QUIP-RS were used as follows: gambling ≥6, sex ≥8, buying ≥8, eating ≥7, combined impulse control behaviors (gambling, sex, buying, eating) ≥ 10, and hobbyism-punding ≥7 (Weintraub et al., 2012). No cut-off for indiscriminate drug use has been established to date (Weintraub et al., 2012). These questionnaires and scales were carried out on PD patients ON medication/ON stimulation. The motor part of the Movement Disorders Society-Unified Parkinson’s Disease-Rating Scale (MDS-UPDRS part III) (Goetz et al., 2008) was used to assess motor impairment. The motor state of the patients and the tasks testing the different types of impulsivity were assessed under four different conditions: ON and OFF dopaminergic medication and ON and OFF stimulation (see below). All participants had normal or corrected-to-normal vision. All participants signed informed consent to participate in the study.

### Impulsivity tasks

#### AX continuous performance task (AX-CPT)

**Figure 1:**
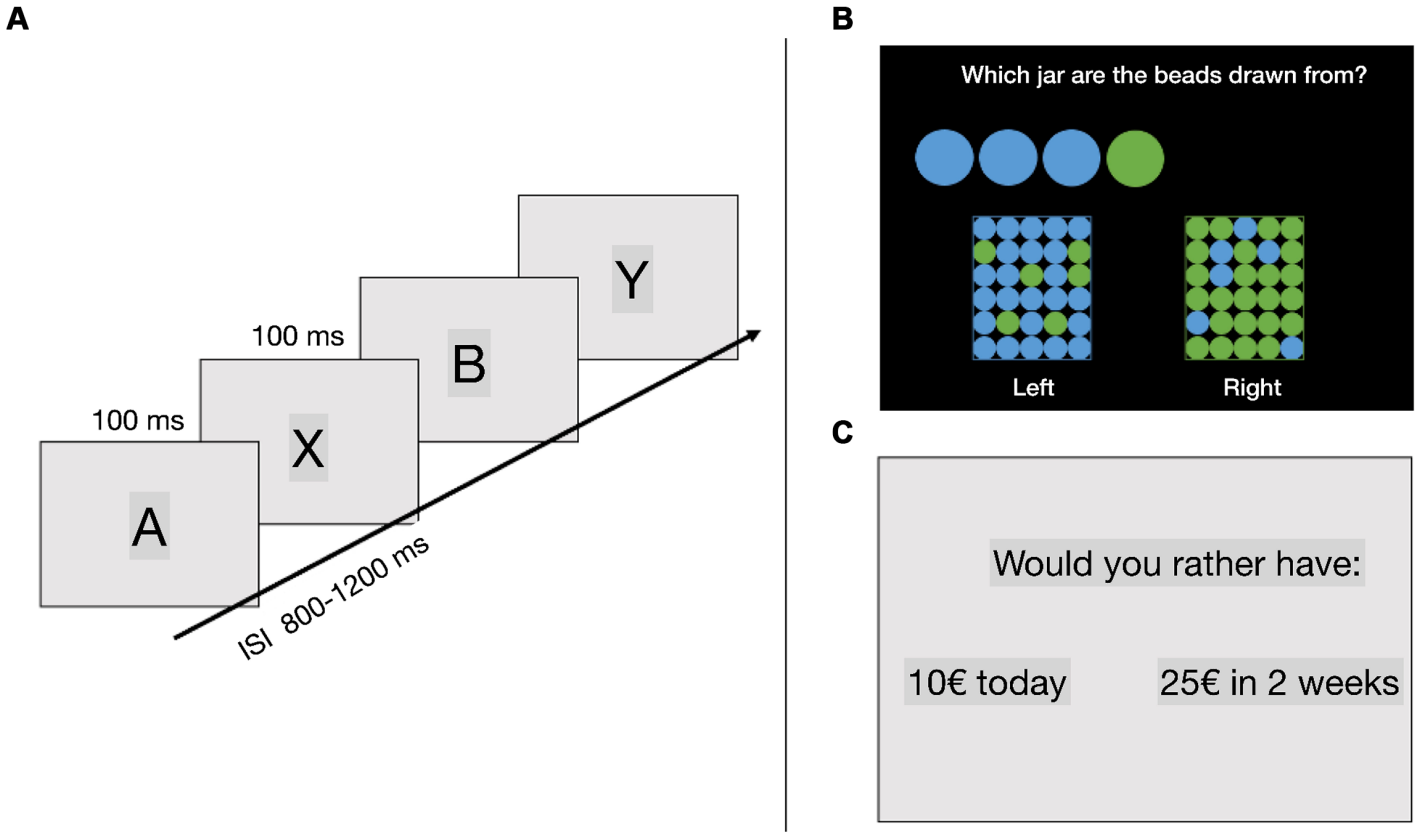
**(A)** AX continuous performance task (AX-CPT), **(B)** beads task, **(C)** delay discounting task. ISI, interstimulus interval.

This task was used to test for motor impulsivity, and it was adapted based on the version used by Gonthier et al. (2016). Different letters were individually presented in the middle of a computer screen in one of the possible orders: AX, AY, BX, and BY. First, a cue letter was presented for 100 ms, followed by an interstimulus interval of 800–1,200 ms. After the interval, a probe letter was presented for 100 ms (Figure 1A). Participants were instructed to indicate only by pressing a key every time an “X” appeared after an “A,” thus making AX trials the “go” trials, and a “no-go” response to all other letter sequences (AY trials: an A cue followed by any probe other than X, BX trials: any cue other than A followed by an X probe, and BY trials: any cue other than A followed by any probe other than X). Therefore, the key variable in the task was the contextual clue, which interacts with target response biases produced by frequency manipulations involving target trials, and three non-target trial types. Following the A-cue context, there is a high expectancy that the trial will require an AX target response. In turn, this target expectancy produces interference when it is not valid, as it occurs on AY non-target trials (where Y refers to any non-X letter). The AY trials allow for reactive control and are composed of a correct cue and an incorrect probe, where the cue letter “A” establishes an expectancy to make a response to the successive probe. Because the successive letter is not “X,” participants need to stop the prepared response (Canário et al., 2019). In contrast, activation and maintenance of the B-cue context (which can be any non-A letter) is critical to overcome the target response bias generated by the X-probe letter on BX non-target trials. The BX trials allow for an analysis of proactive control since the letter “B” serves as a “no go”-cue and prepares the participant to proactively stop their response (Canário et al., 2019). Finally, BY non-target trials provide an internal baseline measure of general performance ability. There were a total of 210 trials, 40% of which were AX trials, 10% were BX trials, 10% were AY trials (A letter followed by a letter other than X), and 40% were BY trials (letter other than A followed by a letter other than X). The experiment consisted of 7 blocks of 30 trials, and the order of trials was randomised across blocks. The main outcome variables in this task were error rates on AX, BX, AY, and BY trials. Reaction time (RT) on AX trials were also recorded.

#### Beads task

This task was used to test for reflection impulsivity. Participants were presented with two jars on the screen, one primarily containing green beads (with fewer blue beads) and the other primarily containing blue beads (with fewer green beads). On each trial, the computer selected one of the jars and the participants started drawing beads from that jar, which were presented on the screen. The participants’ task was to guess to which of the jars the drawn beads belonged (Figure 1B). They could answer whenever they felt that they had enough information to make a guess, a maximum of 9 beads could be drawn on each trial. The ratio in the jars was either 80/20 or 60/40. This task was used as a measure of reflection impulsivity, where less impulsive participants gather more information (draw more beads) before deciding (Djamshidian et al., 2013). The main dependent variable in this task was the number of drawn beads before making a decision.

#### Delay discounting task

This task was used to test for cognitive impulsivity. The task was based on the Kirby test (Kirby and Maraković, 1996). Participants were presented with two options and instructed to choose between them. One option represented a small but immediate reward, while the other option represented a larger delayed reward. For example, participants were offered a choice between “33 € today” and “80 € in 14 days” (Figure 1C). Reward magnitudes (small or high reward) and time delays (from 3 days to 1 year) varied across trials. For each choice, a *k*-value was defined at the indifference level based on a hyperbolic function of delay discounting. For example, a participant with a *k*-value of 0.10 would be indifferent between rewards described in the previous example. The *k*-value can be understood as a measure of cognitive impulsivity, with higher values indicating that individuals place less value on delayed rewards and are more impulsive in their decisions (Kirby et al., 1999). The same number of trials was assigned for each indifferent *k*-value and reward magnitude.

### Procedure

PD patients were tested in four experimental conditions in two sessions, separated by several days: first session involving (1) ON medication/ON stimulation, (2) ON medication/OFF stimulation and second session involving (3) OFF medication/ON stimulation and (4) OFF medication/OFF stimulation. The OFF medication condition was defined as the overnight withdrawal of participants’ regular parkinsonian medication. The OFF stimulation condition was defined as at least 40 min after the DBS was turned off. The participants were tested in the morning on both conditions to minimise any discomfort of being OFF medication and/or stimulation. The order of experimental tasks was counterbalanced between conditions and between patients. Each testing condition started with the assessment of the motor symptoms, followed by the execution of the three experimental tasks. The experimental tasks were presented on a stationary computer using a 27” BenQ LCD monitor positioned 1 m from the participants seated in a comfortable armchair.

### Statistical analysis

SPSS v21 for Mac was used to analyse the data. The Shapiro–Wilk test was used to test the normality of distribution. The data were presented as mean ± SD of the mean and frequency distribution where appropriate. Repeated measures ANOVA with factors condition (four levels) and trial type (two or four levels, depending on the task) was used to analyse the results of the impulsivity tasks between different medication/stimulation conditions in PD patients. A *p*-value of 0.05 was used to denote statistical significance.

## Results

The demographic data and clinical characteristics of the participants are summarised in Table 1. The DBS parameter settings are presented in Table 2. In total, 20 PD patients treated with STN-DBS were included in the study. No patient took extended-release levodopa. Eighteen patients (90%) were taking extended-release dopaminergic agonists in the form of a once-daily morning dose, with a median withdrawal time of 20 h (range 19–24 h). The overnight withdrawal (at least 12 h) was applied for the immediate-release levodopa preparations. Seventeen patients (85%) had BDI-II scores between 0 and 13, indicating no or minimal depression, and three patients (85%) had BDI-II scores between 14 and 19, indicating mild depression. There were no patients with moderate or severe depression. Five patients (25%) reported SAS scores ≥14, indicating apathy, while the rest of the patients had scores lower than 14. Only three patients (15%) had total BIS-11 scores ≥72, indicating impulsiveness; the rest of the patients had total BIS-11 scores lower than 72. No patient had scored above the established cut-offs for gambling, sex, buying and eating. In total, 25% of the patients scored ≥10 on the combined measure (gambling+sex+buying+eating), and 35% had a score ≥ 7 for hobbyism and punding. The overall MDS-UPDRS III score ON medication/ON stimulation was the lowest, while it was the highest OFF/medication OFF stimulation (*F*(3, 57) = 90.29, *p* < 0.001).

**Table 1:** Demographic and clinical characteristics of the Parkinson’s disease patients included in the study [presented as mean ± standard deviation of the mean (SD), frequency, and percentage where appropriate].

		Values	Proportion based on cut-offs	
Age		61.20 ± 8.41	/	
Gender	Male	10 (50%)	/	
	Female	10 (50%)	/	
Years since PD diagnosis		12.72 ± 5.60	/	
Years since STN-DBS		1.90 ± 1.95	/	
MDS-UPDRS-III ON medication	ON DBS	21.55 ± 6.65	/	
	OFF DBS	41.55 ± 8.29	/	
MDS-UPDRS-III-OFF medication	ON DBS	27.45 ± 77.23	/	
	OFF DBS	48.60 ± 11.86	/	
LEDD		627.50 ± 410.56	/	
MoCA		26.35 ± 1.67	/	
BDI-II		8.63 ± 4.12	Mildly depressed	Non-depressed
			3 (15%)	17 (85%)
SAS		9.70 ± 4.14	Apathetic	Non-apathetic
			5 (25%)	15 (75%)
BIS-11 total score		60.30 ± 8.03	Impulsive	Non-impulsive
			3 (15%)	17 (85%)
Attentional BIS-11		9.11 ± 2.13	/	
Motor BIS-11		14.42 ± 3.42	/	
Non-planning BIS-11		23.95 ± 4.46	/	
QUIP-RS total		14.15 ± 9.63		
QUIP-RS gambling		0.15 ± 0.49	Score < 6	Score ≥ 6
			20 (100%)	0 (0%)
QUIP-RS sex		2.15 ± 2.13	Score < 8	Score ≥ 8
			20 (100%)	0 (0%)
QUIP-RS buying		1.60 ± 2.06	Score < 8	Score ≥ 8
			20 (100%)	0 (0%)
QUIP-RS eating		3.20 ± 2.71	Score < 7	Score ≥ 7
			19 (95%)	1 (0.5%)
QUIP-RS combined (gambling+sex+buying+eating)		7.15 ± 4.74	Score < 10	Score ≥ 10
			15 (75%)	5 (25%)
QUIP-RS hobbyism–punding		5.80 ± 4.79	Score < 7	Score ≥ 7
			13 (65%)	7 (35%)
QUIP-RS medication use		1.20 ± 2.62	/	/

PD, Parkinson’s disease; STN-DBS, deep brain stimulation of the subthalamic nucleus; BDI-II, Beck Depression Inventory-II; LEDD, levodopa equivalent daily dose; MoCA, Montreal Cognitive Assessment; SAS, Starkstein Apathy Scale; MDS-UPDRS-III, Movement Disorders Society Unified Parkinson’s Disease-Rating Scale Part III; BIS, Barratt Impulsiveness Scale-11; QUIP-RS, Questionnaire for Impulsive-Compulsive Disorders in Parkinson’s Disease-Rating Scale. BDI-II scores from 0 through 13 indicated no or minimal depression; scores from 14 through 19 indicated mild depression. Patients with SAS values of 14 and above were considered apathetic. Patients with a total BIS-11 score of 72 and above were considered highly impulsive, and patients with BIS-11 scores between 52 and 71 were considered within the normal range of impulsiveness. The following cut-offs used to determine the presence of certain impulsive behaviors based on QUIP-RS were used: gambling ≥ 6, sex ≥ 8, buying ≥ 8, eating ≥ 7, combined impulse control behaviors (gambling, sex, buying, eating) ≥ 10, and hobbyism–punding ≥ 7. No cut-off for indiscriminate drug use has been established to date.

**Table 2:** Deep brain stimulation parameter settings—active contacts, voltage in volts (V), impedance in ohms (Ω), frequency in Hz, and pulse width in microseconds (μs) for the Parkinson’s disease patients participating in the study.

	Active contact left	Voltage (V) left	Impedance (Ω) left	Active contact right	Voltage (V) right	Impedance (Ω) right	Frequency (Hz)	Pulse width (μs)
1	2	2.30	1,297	10	1.50	1,412	130	60
2	2	2.60	1,041	10	2.60	1,242	130	60
3	1	3.00	1,822	10	2.80	1,252	80	60
4	1	2.40	1,157	10	2.80	1,037	130	60
5	2	2.70	1,239	10	2.70	1,320	130	60
6	1	2.40	1,465	10	3.90	998	160	60
7	2	2.30	1,404	9	2.20	943	130	60
8	1	3.00	956	9	2.50	967	130	60
9	1	1.70	1,648	10	3.20	1,141	180	60
10	1	3.80	824	9	3.40	860	100	60
11	1	2.50	1,120	10	3.00	1,045	80	60
12	2	3.80	870	10	2.40	873	60	60
13	1	1.50	1,114	9	3.50	991	120	60
14	1	3.60	1,226	9	2.00	1,303	130	90
15	2	3.00	829	11	3.60	881	120	60
16	1	3.00	1,822	10	2.80	1,252	80	60
17	1	3.30	1,439	10	2.50	1,205	130	60
18	2	3.00	1,113	10	3.00	1,520	100	60
19	2	2.70	1,170	10	3.20	1,114	130	60
20	2	3.40	1,783	9	3.40	1,891	130	60

### AX continuous performance task

The effect of the order of testing (ON vs. OFF medication and ON vs. OFF stimulation) was not significant (*p* = 0.760). The reaction time on AX trials did not differ significantly between different conditions (*F*(3, 57) = 0.36, *p* = 0.777).

**Figure 2:**
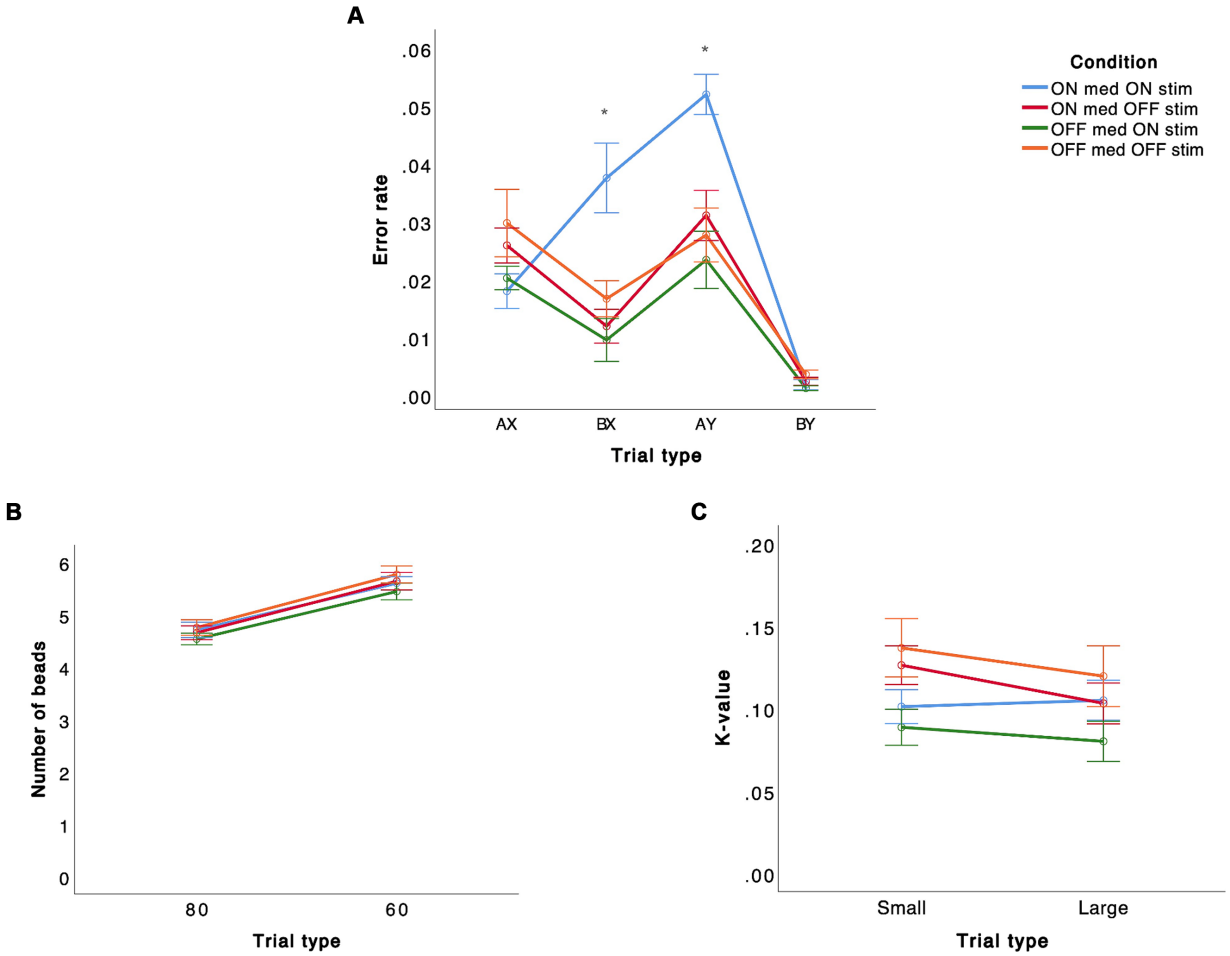
Performance on different tasks [**(A)** AX-CPT, **(B)** beads task, **(C)** delay discounting task] in STN-DBS PD patients in four different conditions are presented. The error bars represent standard deviation of the mean.

There was a significant effect of not only trial type (*F*(3, 57) = 8.918, *p* < 0.001) but also condition (*F*(3, 57) = 3.037, *p* = 0.036) (Figure 2A). This effect was due to higher error rates for both BX (*p* = 0.045) and AY (*p* = 0.006) in the ON medication ON stimulation condition compared to the other conditions. The interaction between trial type and condition was not significant (*p* = 0.065).

### Beads task

The effect of the order of testing (ON vs. OFF medication and ON vs. OFF stimulation) was not significant (*p* = 0.961).

There was a significant effect of trial type (*F*(1, 18) = 56.564, *p* < 0.001) but no significant effect of condition (*p* = 0.691) or interaction between trial type and condition (*p* = 0.887) (Figure 2B).

### Delay discounting task

The effect of the order of testing (ON vs. OFF medication and ON vs. OFF stimulation) was not significant (*p* = 0.346).

There was no significant effect of trial type (*p* = 0.149), condition (*p* = 0.185), or interaction between trial type and condition (*p* = 0.725) for this task (Figure 2C).

## Discussion

Our study examined the effects of dopaminergic medication and STN-DBS on motor, reflection, and cognitive impulsivity domains in PD patients. The main finding of the study was that the combination of dopaminergic medication and STN-DBS had a significant effect on motor impulsivity, increasing both proactive and reactive motor impulsivity subdomains, but had no effect on reflection and cognitive impulsivity domains.

To date, studies have focussed on assessing the effect of dopaminergic medication and STN-DBS alone, showing that either dopaminergic medication (Antonelli et al., 2014; Canário et al., 2019) or STN-DBS (Mirabella et al., 2012; Georgiev et al., 2016) can increase motor impulsivity. Our results are therefore the first to demonstrate that the combination of STN-DBS and dopaminergic medication causes motor impulsivity in STN-DBS-operated PD patients. In addition, the results of our study indicate that both proactive and reactive inhibitory control are impaired in patients ON stimulation and ON medication. While proactive inhibition (BX trials) refers to the ability to prepare for inhibition, involving recruiting the stopping network before inhibition occurs, reactive inhibition (AY trials) does not involve preparation but rather the reactive cessation of a response that has already been initiated (Esteban-Peñalba et al., 2021). Indeed, STN-DBS has previously been shown to be associated with the modulation of networks associated with both proactive and reactive inhibition (Ballanger et al., 2009). These networks include the pre-supplementary motor area, the dorsal and ventral premotor cortex, the dorsal anterior cingulate cortex, the primary motor cortex, and the inferior frontal cortex. Similarly, dopaminergic medication has been shown to impair both proactive and reactive inhibition in PD (Mirabella et al., 2024), although the results of other studies suggest selective dopaminergic medication-dependent impairment of reactive inhibition, and preservation of proactive inhibition (Canário et al., 2019), or conversely an impairment of proactive inhibition and preservation of reactive inhibition (Kricheldorff et al., 2023).

We found no effects of dopaminergic medication and STN-DBS on cognitive and reflection impulsivity. Our results are consistent with data from studies showing no differences in cognitive impulsivity between PD patients and healthy controls (Antonelli et al., 2011), suggesting normal performance of PD patients on cognitive impulsivity tasks. However, some studies have found differences in different subgroups of PD patients. In contrast to patients with PD without impulse control disorders, patients with PD with impulse control disorders show impaired cognitive impulsivity (Rossi et al., 2010). In our study, the rate of impulse control disorders among patients was low, which could explain the relatively stable level of cognitive impulsivity on which dopaminergic medication and STN-DBS had no effect. In PD patients with impulse control disorders, activation of cortical and subcortical areas that play a role in the performance of cognitive impulsivity tasks, such as the ventromedial prefrontal cortex and amygdala-ventral striatum system, has been observed (Rossi et al., 2010). In addition, dopaminergic medication, particularly dopamine agonists, may promote cognitive impulsivity in PD. STN-DBS may also be involved in cognitive impulsivity, in terms of its role in conflictual decision-making, although the evidence for the involvement of STN-DBS in motor impulsivity is much stronger.

Reflection impulsivity, sometimes considered a subtype of cognitive impulsivity, is the least studied form of impulsivity in PD. Similar to cognitive impulsivity, the results on reflection impulsivity tasks may depend on the presence of impulse control disorders (Hlavatá et al., 2019), which could explain our results as the rate of impulse control disorders in our patients was small. However, another study found an effect of dopaminergic medication, particularly dopamine agonists, on reflection impulsivity tested with the beads task even in patients without impulse control disorders (Djamshidian et al., 2013). In this study, patients taking dopamine agonists gathered significantly less information and made more irrational decisions, independent of STN-DBS, suggesting that reflection impulsivity may not be directly dependent on STN activity. Nevertheless, similar to our study, some studies have found no impairment of reflection impulsivity in PD (Czernecki et al., 2002; Euteneuer et al., 2009).

Impulsivity is a complex concept that encompasses various processes and subdomains, including motor, cognitive, and reflection impulsivity. Compared to cognitive and reflection impulsivity, motor impulsivity seems to be better characterised and defined. The STN-DBS may be involved in cognitive and reflection impulsivity in relation to its role in limbic circuity and its role in high-conflict decision-making processes (Antonelli et al., 2011; Jahanshahi, 2013; Jahanshahi et al., 2015). However, the role of STN-DBS in motor impulsivity is much more obvious, although there are still open questions, such as which factors influence STN activity and response threshold adaptation (Jahanshahi et al., 2015). The predominant involvement of the STN in motor impulsivity may partly explain our results showing a clear effect of STN-DBS (and dopaminergic medication) on motor but not on cognitive and reflection impulsivity. Furthermore, the variability of results across studies could be due to the fact that different studies used different paradigms to explain the same or a similar phenomenon (e.g., Go-No-Go and Stop-signal task to explain motor impulsivity) (Jahanshahi, 2013; Jahanshahi et al., 2015). In addition, the study populations also differ significantly in terms of the degree of impulsivity of the study population, which could influence cognitive and reflection impulsivity, especially in impulse control disorders. In our study, a small proportion of patients were categorised as impulsive based on either the BIS-11 or the QUIP-RS, which could explain the fact that dopaminergic medication and STN-DBS did not affect these subdomains of impulsivity.

The main limitations of our study were the relatively small sample size and the fact that we did not include healthy controls. However, the PD patients in our study were tested under four different conditions to investigate the effect of dopaminergic medication and STN-DBS on three different types of impulsivity. By comparing different treatment conditions, including both DBS and dopaminergic medication, in the same patients, we were able to reduce potential differences between patients. In addition, the main objective of the study was to test the effect of dopaminergic medication and STN-DBS on different domains of impulsivity in PD. Our study focussed on short-term changes in treatment conditions. However, we used the common real-world definition of nocturnal drug withdrawal to define the OFF state in PD. In addition, we wanted to test the acute effect of stimulation manipulations on impulsivity. Although a 40-min stimulation withdrawal may seem short, there was a clear difference in motor state with stimulation OFF versus simulation ON, both with dopaminergic medication ON and OFF. In addition, a relatively short period of stimulation withdrawal has been used to date in many studies (Pillon et al., 2000; Plessow et al., 2014; Brandt et al., 2015; Castrioto et al., 2015). PD patients included in our study did not have dementia. This fact allowed for greater power to detect differences between conditions. However, future studies would benefit from including patients with different stages and severity of cognitive impairment that could allow for the evaluation a possible relationship between impulsivity and cognitive impairment or impairment of individual cognitive domains. We found an effect of stimulation and medication on motor impulsivity but not on cognitive and reflection impulsivity. Future studies might benefit from using more than one task for different types of impulsivity, although this could significantly increase the duration of experiments to ensure that the lack of effect is truly due to the lack of effect of medication and/or stimulation on specific domains of impulsivity rather than the inability to detect the effect due to task-related characteristics.

In summary, our study found that the combination of dopaminergic medication and STN-DBS leads to increased motor impulsivity but does not affect cognitive and reflection impulsivity in PD patients. This finding could have clinical implications by highlighting the potential worsening of impulsivity when combining dopaminergic medication and STN-DBS. Future research examining the effects of dopaminergic medication and STN-DBS is needed to replicate our findings and to focus on long-term differences in different domains of impulsivity.

## Data availability statement

The datasets presented in this article are not readily available because the data that support the findings of this study are available from the corresponding author, DG, upon reasonable request. Requests to access the datasets should be directed to dejan.georgiev@kclj.si.

## Ethics statement

The studies involving humans were approved by Medical Ethical Committee of the Republic of Slovenia. The studies were conducted in accordance with the local legislation and institutional requirements. Written informed consent for participation in this study was provided by the participants' legal guardians/next of kin.

## Author contributions

MH: Data curation, Investigation, Visualization, Writing – original draft, Writing – review & editing. SV: Validation, Writing – review & editing. RB: Validation, Writing – review & editing. MB: Writing – review & editing. MJ: Validation, Writing – review & editing. MT: Validation, Writing – review & editing. DG: Conceptualization, Data curation, Formal analysis, Funding acquisition, Investigation, Methodology, Supervision, Validation, Visualization, Writing – review & editing.
